# Generation of stable ARE- driven reporter system for monitoring oxidative stress

**DOI:** 10.1186/s40199-015-0122-9

**Published:** 2015-08-01

**Authors:** Paria Motahari, Majid Sadeghizadeh, Mehrdad Behmanesh, Shaghayegh Sabri, Fatemeh Zolghadr

**Affiliations:** Faculty of Biological Sciences, Department of Molecular Genetics, School of Biological Sciences, Tarbiat Modares University, Jalal Ale Ahmad Highway, PO Box 14115–111, Tehran, Iran; Department of Medical Genetics, School of Medical Sciences, Tarbiat Modares University, Tehran, Iran

**Keywords:** Antioxidants, Nrf2-ARE, Oxidative stress, Reporter cell line

## Abstract

**Background:**

NF-E2-related factor2 (Nrf2)-antioxidant response element (ARE) signaling pathway is the major defensive mechanism against oxidative stress and is up regulated by specific antioxidants and oxidants to comprise the chemoptotective response. Detection of ARE-activating compounds helps to develop new drugs and identify/quantify the tension range of the oxidants.

Important reasons promoting this work are high throughput, rapid and inexpensive experiments relative to the in vitro studies for ARE-Nrf2 pathway monitoring of chemicals and environmental samples.

**Methods:**

In this study hepatoma Huh7 reporter cell line was generated which contains a luciferase gene under the control of an ARE. This is the first example of ARE construct containing one copy of extended consensus response element. The cells were treated with hydroquinone (HQ) and p-benzoquinone (BQ) (oxidative stress inducers) and the antioxidant, curcumin.

**Results:**

The luciferase activity was induced in a concentration-dependent manner in a concentration range of 1–2 μM for BQ and HQ. Curcumin was also validated as an ARE inducer in concentration above 10 μM. In addition, this reporter cell line provides a rapid detection as early as 4 h to respond to the ARE inducers.

**Conclusion:**

It is a powerful tool for the sensitive and selective screening of chemicals, drugs and environmental samples for their antioxidant and oxidant activities.

## Background

Monitoring compounds on pathways of interest to provide insight into the molecular mode of action to evaluate the possible toxicity is required in modern medicine. Furthermore, the lack of information about the cellular signaling pathways of drugs is a weakness in clinical treatment. Recently in vitro studying of pathways involved in genotoxic responses is developed to determine whether specific compounds affecting particular pathways [1]. Due to environmental issues, antioxidant and oxidant response pathway has attracted much more attention [2]. Reactive oxygen species (ROS) are highly unstable molecules; interfering with the normal cell function by trigging cascade of oxidative stress pathways. Defensive system in response to oxidative tension turns multifarious of signaling pathways on to protect the cells [3]. Mounting evidence demonstrated that human genome consists of protective genes acting through the enhanced sequence known as the ARE in their promoter region. Metabolization by the ARE mechanism is one of the common responses, which is also induced by the certain groups of antioxidants to confer guard against oxidative tension [4]. The regulation of these genes is performed by Nrf2 transcription factor (NF-E2-related factor2). Heterodimerized Nrf2 along with other factors including Jun and small Maf proteins binds to the ARE leading to the expression of ARE target genes [5].

The aim of this study was to establish a sensitive, stable ARE reporter cell line, using a luciferase gene under the transcriptional control of one copy of the ARE core sequence of the human NQO1 promoter (NAD (P) H: quinoneoxidoreductase) (one of the Nrf2 target genes) and it was carried out using immortalized cells derived from human liver. The reporter cell line was challenged with a range of compounds, to evaluate the ARE activation potential in the engineered cell line. The reporter cell line is sensitive enough to screen chemicals with toxicity risk and drugs with antioxidant activity related to the activation potency of the ARE signaling pathway.

## Methods

### Chemicals

Fetal bovine serum (FBS) was purchased from Invitrogen/Life Technologies (Carlsbad, CA, USA). BQ, HQ, dimethyl sulfoxide (DMSO) were the products of Sigma-Aldrich (St. Louis, USA). Curcumin was purchased from Merck KGaA (Darmstadt, Germany) with a purity of 95 %.

### Cell culture and condition

The human hepatoma Huh7 cell line was purchased from National Cell Bank, Pasteur Institute of Iran and were grown in Dulbecco’s modified Eagle’s medium (DMEM) (Invitrogen/Life Technologies,Carlsbad, CA, USA). Cells were supplemented with 10 % fetal bovine serum, 100 U/mL penicillin, and 100 mg/mL streptomycin (Invitrogen/Life Technologies, Carlsbad, CA, USA). Cells were grown at 37 °C in a humidified atmosphere of 5 % carbon dioxide. Cells tripsinized/sub cultured every 2 to 3 days.

### Oligonucleotides

The NQO1 ARE was originally described by Li and Jaiswal in a 5′-TCG AGA TGC AGT CAC AG**T GAC TCA** GCA GAA TCT GA-3′ and 3′-CTA CGT CAG TGT CAC TGA GTC GTC TTA GAC TAG CT-5′ nucleotides sequences (Activator protein 1 (AP1) binding site is shown in bold) [6]. ARE oligonucleotides were commercially synthesized through *XhoI*-*HindIII* restriction sites at either ends (oligonucleotides were synthesized by Bioneer (Daejeon, South Korea). The synthetic oligonucleotides were annealed, phosphorilated, purified on a 12 % polyacrylamide gel and cloned at the *XhoI*-*HindIII* sites of pGL4.26 using standard protocols. The orientation and sequences of this element were confirmed by sequencing of the plasmids.

### Chemicals exposure

Sterile stock solutions of BQ, HQ and curcumin were prepared in DMEM just before use. Briefly, Cells were seeded at a density of 2 × 10^5^ per well in 24-well microtiter plates, and incubated until cells reached 70–80 % confluence. Following overnight recovery, the culture medium was replaced by the fresh DMEM supplemented with antibiotics along with a range of chemical concentrations in triplicate for 4 h, 6 h, 8 h and 24 h to estimate the luciferase shortest induction time.

### Cell viability assay

Cell viability was assessed by methylthiazoltetrazolium (MTT) assay (Sigma-Aldrich, St Louis, USA) according to the manufacturer’s instruction. Briefly, cells (1 × 10^4^) were cultured overnight in a 96-well plate. Afterwards, the medium of each well was replaced by 200 μL fresh medium plus 50 μL of the MTT solution (5 mg/mL in PBS). The plates were incubated at 37 °C for 4 h. The absorbance being proportional to cell was subsequently measured at 570 nm in each well using an enzyme-linked immunosorbent assay plate reader (Bio-RAD 680, USA). All experiments were performed in triplicate, and the relative cell viability (%) was calculated as a percentage relative to the untreated control cells.

### Development of an ARE luciferase-reporter

**Fig. 1:**
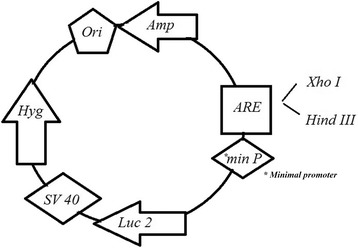
A schematic representation of pGL4.26-1x-ARE construct. ARE was cloned at the *XhoI*-*HindIII* sites of pGL4.26 upstream of the firefly luciferase gene

The ARE-luciferase reporter plasmids were generated using the pGL4.26-minimal promoter vector (Promega, UK, Southampton, United Kingdom) containing a minimal TATA promoter upstream of the firefly luciferase gene. Double-stranded oligonucleotides ligated into the pGL4.26 [minP]. Consequently, TOP10 competent cells were transformed with the recombinant DNA for amplification. Engineered vector contains one copy of ARE sequence that have been inserted, in head-to-tail orientation, through *XhoI*-*HindIII* restriction sites upstream of the promoter-luc + transcriptional unit (Fig. 1). Eight positive clones were sequenced using the RV primer.

### Huh7-1x-ARE-luc reporter-gene assay

Huh7 cells were seeded in 24-well plates at a density of 2 × 10^5^ cells per well and grown overnight. Consequently, the cells were transiently transfected with the recombinant reporter plasmids. The plasmid pGL4.26 without the ARE piece, was considered to control the performance of the construction. Transfection was done by LipofectAMINE 2000 (Invitrogen, Carlsbad, CA, USA) reagent in triplicate according to the manufacturer’s instructions. Following transfection the culture medium was replaced 24 h later with the fresh growth medium containing 10, 20, 30 μmol/L HQ which was prepared immediately before each experiment. Cells were left for 24 h to respond the oxidative stress inducers, and then the firefly luciferase activities in their lysates were monitored.

### Luciferase assay

Luciferase assays were performed following the manufacturer’s instruction (Promega). Cells were washed twice with phosphate buffered saline (PBS). Each well received 75 μl lysis buffer (Promega, UK, Southampton, United Kingdom) after removing PBS. Cell lysates were harvested and spin for 5 min. The cell lysates (50 μl) were added to 100 μl luciferase assay reagent (Promega, UK, Southampton, United Kingdom). Luciferase bioluminescence measurements were performed at room temperature using a luminometer (Sirius tube Luminometer, Berthold Detection System, Germany). Activity was expressed as relative light units (RLU) emitted from total assays and it was calculated versus background activity.

### Generation of stable ARE-driven reporter cell line

The Huh7-1x-ARE-luc containing the hygromycin selectable marker was stably transfected into the Huh7 cells using the LipofectAMINE 2000 reagent. According to the LD50 value transfected cells were selected using 450 μM hygromycin (Invitrogen/Life Technologies, CA, USA) in the media for 4 to 5 weeks. The hygromycin-resistant clones were isolated and screened by measuring their basal and inducible luciferase activity at different concentration of HQ and BQ. Positive clones, which showed high inducible luciferase activity, were passaged and maintained in growth medium containing 450 μM hygromycin for further analysis.

### Statistical analysis

All experiments were conducted in triplicate, and the results were expressed as mean ± SD (standard deviation). One-way analysis of variance (ANOVA) was used to assess the statistical analysis, and *p* <0.05 was considered to be significant*.* Data was analyzed using Microsoft Excel software and GraphPad InStat software.

## Results

### Cytotoxic studies

**Fig. 2:**
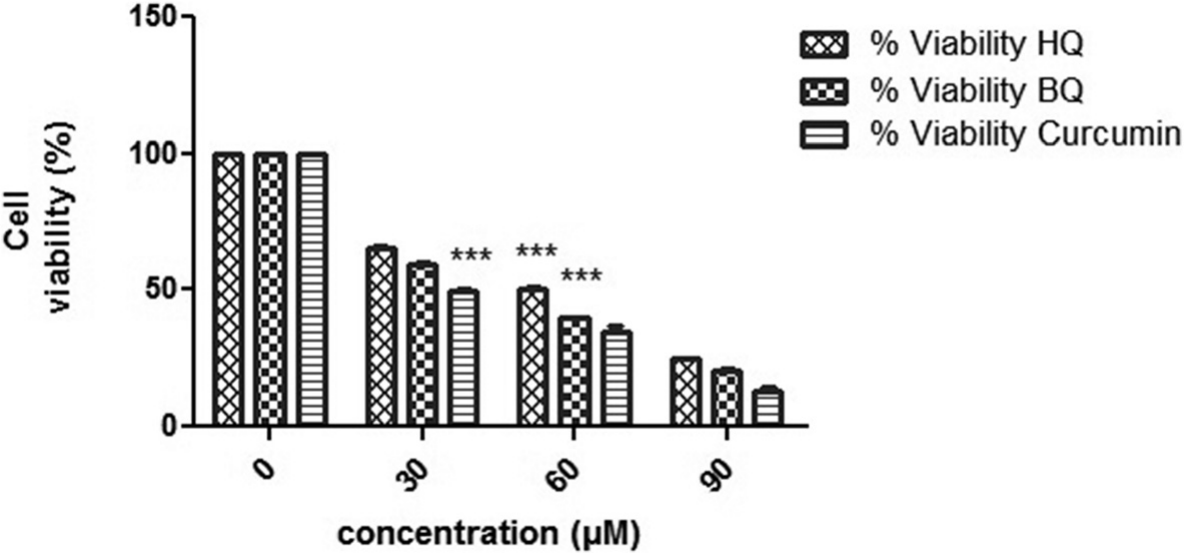
Toxicity of HQ, BQ and curucmin to human hepatoma (Huh7) cells. Huh7 cells were cultured with different doses for 24 h as indicated in the Methods. Left column represents the percentage of cell viability treated with chemicals. Data showed LD50 values of 60 μM HQ and 45 μM BQ and 30 μM curucmin for Huh7 cells. Notes: Data expressed as mean ± SD; ***, *p* <0.001 compared to nontreated cells

Data from MTT assay clearly showed LD50 values of 60 μM HQ and 45 μM BQ and 30 μM curucmin for huh7 cells and demonstrated higher levels of either BQ, HQ or curucmin inhibits cell metabolism of Huh7 cells. (Fig. 2)

### Transient transfection and analysis of luciferase reporter gene activity

**Fig. 3:**
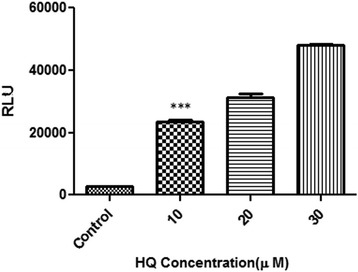
Luciferase reporter activity in transiently transfected Huh7-1x-ARE-luc cells. Recombinant cells were seeded overnight in 24 well plates at 2 × 10^5^ cells per well. Cells were incubated with increasing concentration of HQ (10, 20, and 30 μM). After 24 h of treatment, luciferase activity was assessed by measuring luciferase activity in cell lysates. Control cells (which lacks any ARE) failed to induce luciferase activity. Notes: Data expressed as mean ± SD; ***, *p* <0.001 compared to control

Prior to stable transfection, ARE responsiveness of the plasmid pGL4.26-1x-ARE-luc was confirmed in transient transfection experiments and it was found that stimulation of ARE could induce expression of luciferase in these cells (Fig. 3). HQ treatment of Huh7 cells transfected with pGl4.26-luc (which lacks any ARE) failed to induce luciferase activity, whereas a significant induction of luciferase activity was observed in cells transfected with pGl4.26 -1x-ARE-Luc. HQ (10 μM) caused an approximately 11-fold increase in luciferase activity compared to the negative control during 24 h of exposure (*p* <0.001).

### Generation of a stable ARE reporter cell line and oxidative stress induction assay

**Fig. 4:**
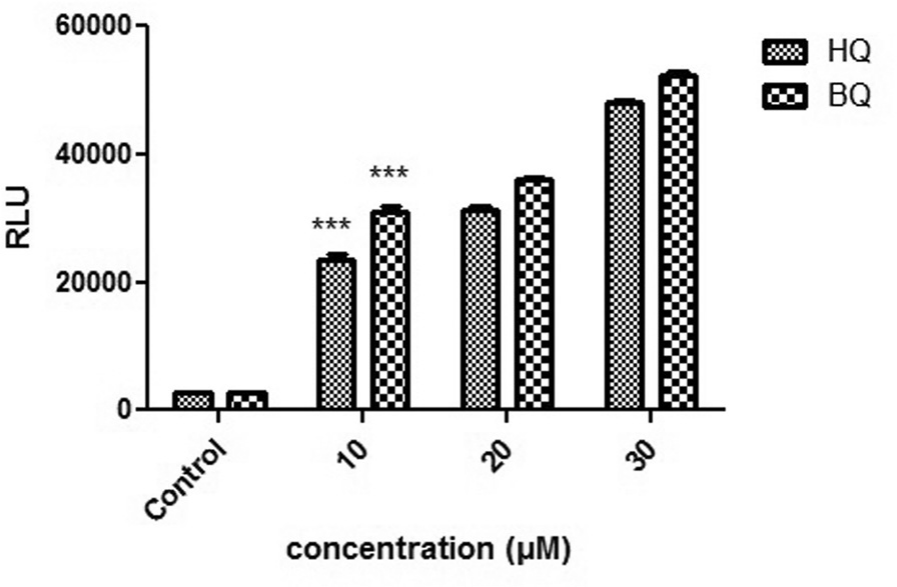
ARE inductions in stable transfected Huh7 exposed to HQ and BQ (10, 20, and 30 μM). Luciferase activity was measured after 24 h. The numbers in the left column represent the relative luciferase activities. Luciferase activity was increased following treatment with increasing concentrations of oxidative stress inducers. Each bar shows the mean ± SD; ***, *p* <0.001 compared to control

The pGL4.26-1x-ARE-luc plasmid containing the hygromycin selectable marker was stably transfected into Huh7 cells. Cells were selected for ARE inducibility in media containing 450 μM hygromycin for 4 weeks (according to the hygromycin LD50, data are not shown). Stable clones were isolated and screened by measuring their inducible (obtained by treatment with 10, 20 and 30 μM BQ, HQ) luciferase activities as described before. As mentioned in Fig. 4 luciferase activity was increased following treatment with different dosage of oxidative stress inducers (*p* <0.001).

No changes in the ARE responsiveness of Huh7-1x-ARE-luc cells were measured over eight passages and after multiple rounds of storage in liquid nitrogen and re-culture.

### Dose dependent of luciferase induction by oxidative stress inducers in the recombinant cell line

**Fig. 5:**
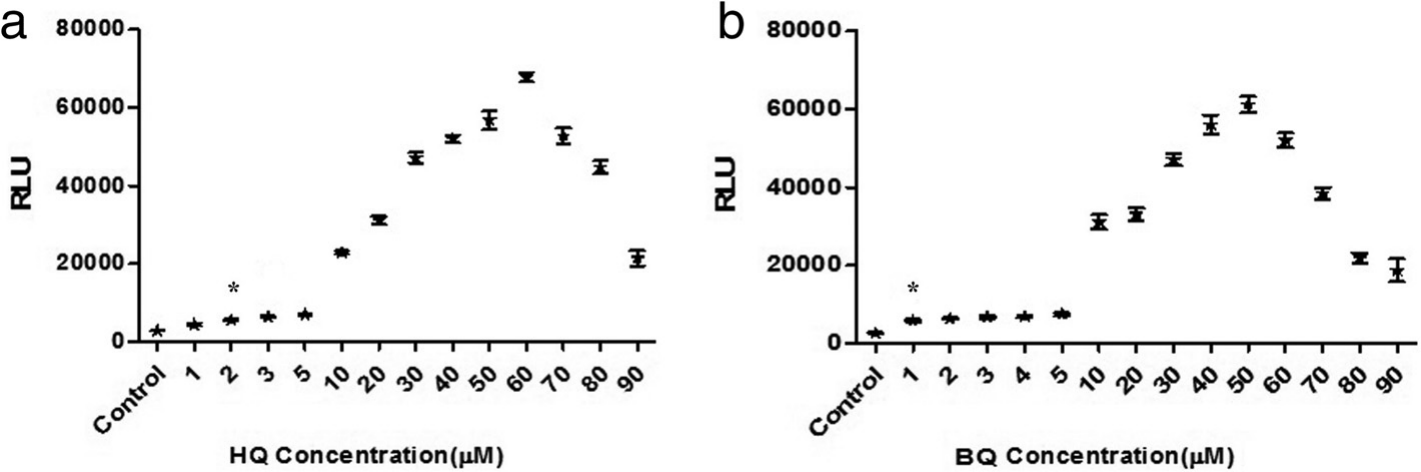
Induction of luciferase activity in a dose-dependent manner. Cells were incubated with increasing concentration of HQ(**a**) and BQ(**b**). Maximal stimulation reached at 60 μM of HQ and 50 μM of BQ. Around 2.5 fold increases luciferase activity was seen following treatment with 1 μmol/L BQ and ~ 2.4 fold by 2 μmol/L HQ. The results are presented as the mean ± SD; *, *p* <0.05, as determined by one way ANOVA, compared with the corresponding no treated control

When it was proved that the recombinant cells were sensitive to ARE stimuli treatment, the dose dependent effects of inducers on this cell line was investigated. In this regard cells were incubated with increasing concentrations of BQ and HQ. Inducers stimulated luciferase activity in a dose dependent manner, with maximal stimulation reached at 60 μM of HQ and 50 μM of BQ (Fig. 5). Furthermore a minimum luciferase activity (around 2.5 fold increases) was seen following treatment with 1 μmol/L BQ and ~ 2.4 fold by 2 μmol/L HQ (*p* <0.05).

### Shortest induction validation

**Fig. 6:**
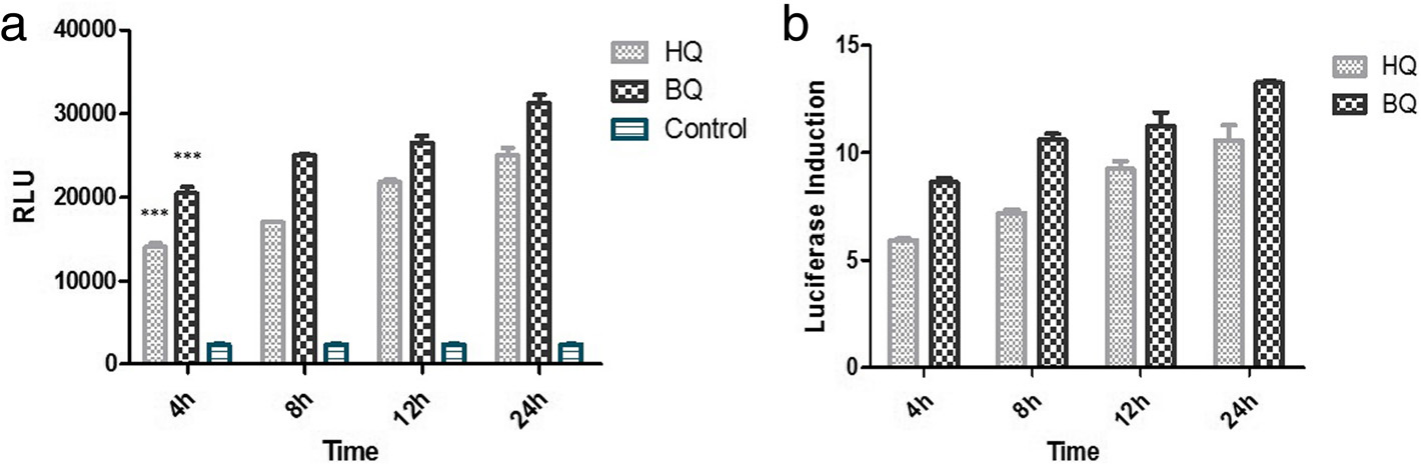
The time course of luciferase activity induction. The Recombinant cell line was incubated with HQ and BQ (10 μM) at 37 °C for the indicated time after which luciferase activity in cell lysates was measured as described under the “Methods” section. Luciferase activity was expressed as (**a**) RLUs or (**b**) as a percent of the luciferase induction by HQ and BQ in recombinant Huh7 cell line. The luciferase induced significantly after 4 h as the shortest induction time. Each bar shows the mean ± SD; ***, *p* <0.001 compared to control

Induction of luciferase activity by BQ and HQ was also time dependent; it increased 6-fold after four hours, 7.3-fold after eight hours, 9.1-fold after twelve hours of treatment with 10 μmol/L HQ and reached 10-fold after 24 h of treatment with the same dose of HQ. The luciferase induced significantly after 4 h as the shortest induction time (*p* <0.001). The time course of induction of luciferase by 10 μM of HQ and BQ in recombinant cell lines is shown in Fig. 6.

### Huh7- 1x-ARE-luc sensitivity to antioxidants

**Fig. 7:**
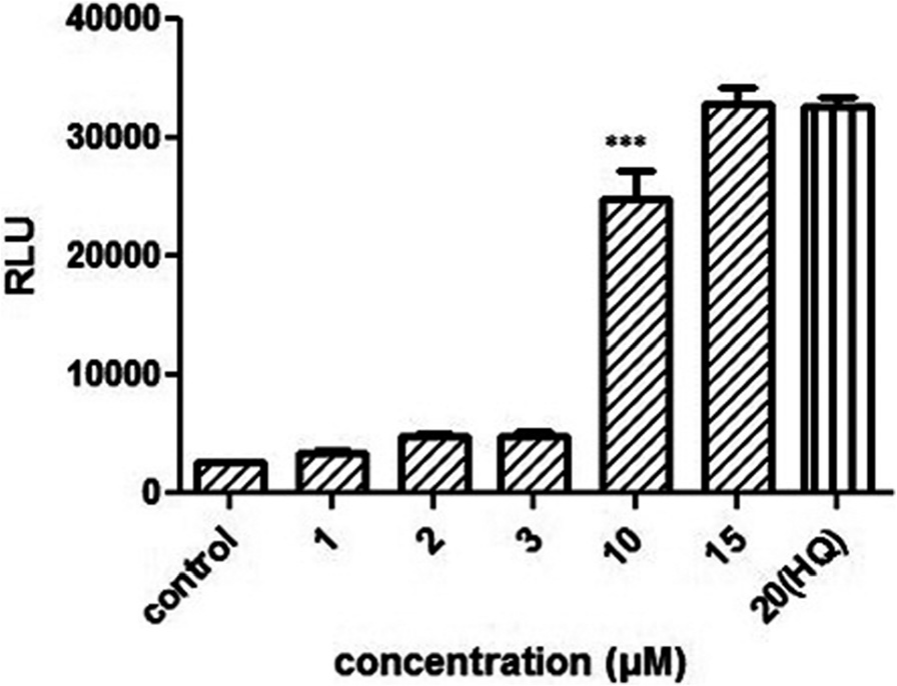
ARE induction in Huh7-1x-ARE-luc after exposure to 1, 2, 3, 10, 15 μM of curcumin. Briefly, Cells (2 × 10^5^) were cultured overnight in a 24-well plate. Luciferase activity was measured 24 h after exposure to different concentration of curcumin. (***, *p* <0.001)

Huh7-1x-ARE-luc cells were subsequently assessed to examine whether recombinant cells would also respond to compounds with antioxidant activity through ARE mediated activation. In this regard, luciferase activity induction was tested after recombinant cell line treatment with curcumin (diferuloylmethane or 1-7-bis (4-hydroxy-3-methoxyphenol)-1,6-heptadiene-3,5-dione). ARE induction by curcumin in recombinant cells was observed after incubation for 24 h, and the induction effects were detected at 10 μM concentrations of curcumin (*p* < 0.001)(Fig. 7). In contrast, low concentration of curcumin did not induce luciferase activity.

## Discussion

Possible threat of food and environmental compounds to human health through elevation of oxidative stress level shows the importance of ARE induction monitoring after exposure with suspected compounds. Studying Nrf2-ARE signaling pathway of drugs and natural compounds with antioxidant activity gives target information to discover novel drug-protein relationship [7].

In this study a stable ARE- reporter cell line was generated; it provides a good model system to screen and identify chemicals as ARE inducers and explores induced pathway of natural and synthetic antioxidants. The engineered cell line called Huh7-1x-ARE-luc, contained the 31 nucleotides core promoter of human NQO1 (Nrf2 targeted gene) ARE, which is used to direct expression of luciferase. Primitive investigation showed a necessary core sequence of ARE as a sufficient sequence to mediate induction [6]. Further nucleotide sequence analysis in the human NQO_1_ gene ARE (hARE) revealed the requirement for perfect AP1 and imperfect AP1 elements arranged in inverse orientation at the interval of three base pairs and a GC box for optimal expression. Huh7-1x-ARE-luc contains APl binding site (5′-TGACTCA-3′) and imperfect AP1 (5′-ACTGACG-3′) and nucleotides GCA (GC box) located within the human ARE considered to be responsible for more transcription of the respective genes [8]. The stable cell line have the benefit of high correlation with Nioi new reported ARE sequence (5′-gagTcA**C**aGTgAGt**C**ggCAaaatt-3′) which two cytosine (shown in bold) residues are substituted with two ‘n’ residues [9]. Thus far no other ARE cell lines have been shown to match with the new reported sequence and just limited to TMAnnRTGAYnnnGCRWWW core promoter. This cell line has the potential to be a unique tool because it is the first example which has used huh7 cell line. The main advantages of using HuH7 cell line for research is that, sharing several characteristics of normal hepatocyte. These cells offer stable phenotype, reproducibility and cheaper to culture, which make them useful for in vitro drug safety analysis [10]. Human hepatoma Huh-7 cell line is a proper model system to study liver metabolism and toxicity of xenobiotics; and the detection of antitoxic agents [11]. One characteristic of Huh7 cells that makes them attractive for use them in toxicity studies is the expression of drug metabolizing enzymes. These cells display the functional activities of various carbohydrate-metabolizing enzymes and represent an alternative model system for drug efficacy or toxicity studies related to the liver-specific genes induction [12]. In vitro liver cell line are increasingly used for the toxicological studies due to the central role of the liver in chemical transforming and clearing.

The performance of this assay was evaluated using HQ and BQ as oxidative stress inducers and curcumin as a compound with antioxidant activity. HQ and BQ are metabolites which derived from benzene biotransformation in liver and have been shown to produce reactive oxygen species; and causing oxidative stress [13]. Furthermore, a number of protective mechanisms; via the ARE are induced in response to HQ and BQ cytotoxicity [14, 15]. So these are a good choice to challenge the construct to explore sensitivity. The responsiveness of this stable cell line to BQ and HQ is in the 1–2 μM range, which would imply sufficient sensitivity; because the reporter gene expression in pGL4 vectors are increased compared with the other vectors. Even though other studies show that 1 μM of HQ could induce NQO1 activity and less concentration (0.1 μM) could not induce NQO1 [14].

It is shown that luciferase activity in Huh7-1x-ARE-luc cells is dose dependent, this cell line gives a ~ 26 fold increase in reporter gene activity in the presence of 60 μM of HQ and an approximate 25 fold increase in luciferase activity by a 50 μM dose of BQ as the oxidative stress inducers. According to the LD50 values (The LD50 of HQ and BQ following a 24-h exposure was approximately 60 μM and 45 μM, respectively) maximal detection depends on the toxicity. Previously, a stable reporter MCF7 (human breast adenocarcinoma cell line) cell line was generated. The reporter construct contained eight copies of ARE containing promoter sequence from mouse gsta1 and gave 50-fold induction according the treatment with 50 μmol/L t-BHQ [2]. In this study it is shown that single copy of ARE is sufficient to confer responsiveness for a sensitive detection.

As demonstrated, ARE expression reflected by luciferase reporter is in a time-dependent manner. To examine the shortest period induction, luciferase activities were analyzed in 4 h, 8 h, 12 h and 24 h. These experiments revealed that Huh7-1x-ARE-luc could stimulate a small, but apparent induction of luciferase activity as early as 4 h after treatment as a rapid detection.

The construct was also validated with curcumin, a polyphenol yellow pigment in the rhizome of *Curcuma longa Linn* (Zingiberaceae) [16], which is reported to induce Nrf2 in HUh7 human hepatoma cells to exert antioxidant effect [17]. As Rachana Garg and his collogues showed in mice treated with curcumin the level of NQO1 expression increased due to nuclear translocation of Nrf2 and then association with ARE sequences [18]. According to this information, reporter cell line was challenged with curcumin. Huh7-1x-ARE-luc is induced by 10 μM of curcumin. It was demonstrated that less concentration of curcumin could not induce luciferase expression via ARE induction; maybe due to the fact that the antioxidant activity of curcumin through ARE is dose dependent. In this regard, other experiments also revealed that concentration above 10 μM of curcumin increased the expression of genes involved in ARE-Nrf2 pathway in renal epithelial cell [19].

## Conclusion

In vitro assays are becoming more attractive as screening tools because they are rapid, and they have the potential to reduce the number of animals needed for chemical testing. Stably transfected cell lines offer several advantages in comparison to other in vitro systems. They are an excellent aid in defining the mechanism of unknown compounds [20]. Huh7-1x-ARE-luc assay demonstrated the values of an in vitro screen for a battery of tests to examine ARE inducers like oxidative stress stimuli and natural/chemical agents with antioxidant activity. It is a reproducible and reliable in vitro assay that measures the activation of the ARE via a luciferase reporter gene.

## Abbreviations

ARE: Antioxidant response element
BQ: p-benzoquinone
HQ: Hydroquinone
NQO1: NAD (P) H dehydrogenase [quinone] 1
Nrf2: NF-E2-related factor 2
